# Nursing students’ perceptions of soft skills training in Ghana

**DOI:** 10.4102/curationis.v40i1.1677

**Published:** 2017-09-22

**Authors:** Luke Laari, Barbara M. Dube

**Affiliations:** 1 Presbyterian Nurses Training College, Ghana; 2 School of Nursing and Public Health, College of Health Sciences, University of KwaZulu-Natal, South Africa

## Abstract

**Background:**

The quality of nursing care rendered today is markedly reducing and the amount of time spent with patients listening to and explaining issues concerning their conditions is gradually diminishing. The therapeutic touch and the listening ear of the nurse are no longer accessible to the patient. Understanding what non-technical skills are and their relevance for healthcare practitioners has become a new area of consideration. Although recent literature has highlighted the necessity of introducing soft skills training and assessment within medical education, nursing education is yet to fully embrace this skills training.

**Objectives:**

The aim of this study was to explore nursing students’ understanding of the concept of soft skills and to acquire their perception on the need for soft skills training to promote quality nursing care.

**Methods:**

A quantitative research design with descriptive and explorative strategies was used. One hundred and ten nursing students were sampled after permission to conduct the study was requested and obtained from the University of KwaZulu-Natal Ethics Committee.

**Results:**

The results indicated that a majority (68.8%) of respondents understood the concept of soft skills and agreed with the definition of ‘soft skills’. They furthermore agreed that soft skills should be part of the training that student nurses receive during their professional training.

**Conclusion:**

The study revealed that there is a need for nursing students to be educated in soft skills and that this will enhance their job performances in the clinical environment and improve the way in which they communicate with their clients.

## Introduction

Understanding what non-technical or soft skills are and their relevance for healthcare practitioners has become a new area of consideration. Although recent literature has highlighted the necessity of introducing non-technical skills training and assessment within medical education, nursing education is yet to fully embrace this skills training (Pearson & McLafferty 2011:399).

According to Heckman and Kautz (2012:451), soft skills are also known as people’s skills. In their study, ‘Soft Skills or Hard Cash’, they define soft skills as interpersonal qualities and personal attributes that one possesses (Lazarus 2013:40; Ten Hoeve, Jansen & Roodbol 2014:295).

Bramhall (2014:53) and Jootun and McGhee (2011:40) argue that nursing is transforming with the emerging technological advances in the world, but contend that nursing care is deteriorating and may be departing from the provision of quality nursing care. Furthermore, they state that the public outcry about nurses’ poor interpersonal and listening skills towards clients at work is deafening.

According to Derbyshire and McKenna (2013:305), one cannot be unmindful of the outwardly endemic erosion of caring and compassion in nursing. They argue that this crisis has been fermenting for a long time, but only recently came to a head in the United Kingdom. They pose significant questions in their study: what are nursing education’s responsibilities related to the devaluation of fundamental nurse caring and restoration of public confidence in nursing as an elementally caring service? What role has nurse education played in devaluing and marginalising such care? What have we done or failed to do in our curricula, in our teaching or in our relationships with ‘service partners’ that contributed to the situation? According to them, there has been little intervention, except the simplistic sloganeering of getting nursing out of the universities back into the hospitals, and no calls for action have been witnessed directed towards nursing education.

Griffiths et al. (2012:121) in an article ‘A Caring Professional Attitude’ stated that their participants expressed concerns about whether the educational preparation of nurses can develop these caring qualities. It is an undeniable fact that nursing education is supposed to do this relentlessly, but this is not seen explicitly in the nursing curriculum, especially in Ghana. Literature suggests nurses do not communicate as well as they might in healthcare settings (Bach & Grant 2015:208; Bramhall 2014:53; Tuohy 2008:1680). Interpersonal and inter-professional communication and listening skills, among others, are very important in the nursing profession. Essential communication skills are deemed to be listening and attending, empathy, information giving and support in the context of a therapeutic relationship. The focus needs to be person-centred rather than nurse- or task-focused, and the relationship is a key element. Time spent developing this key relationship is an investment and yet is often a precious commodity. Busy wards with high-dependency patients are the norm in many acute care settings, and any time spent in understanding the patient’s individual needs is crucial (Timmins 2011:30). There is some evidence to suggest that, while qualified nurses often rate their own communication skills as high, patients report less satisfaction and maintain that communication could be improved (McCabe & Timmins 2013:130). This suggests that what nurses perceive about their communication skills might not be parallel to the client’s perception. Therefore, it is imperative to balance the scale and determine what exactly the levels of verbal communication, listening and interpersonal skills are.

With expanding immigration, increasing globalisation and minority population growth, there is a need to enrich diversity management within the nursing profession to better meet the needs of our changing society (Bednarz, Schim & Doorenbos 2010:253). According to Bach and Grant (2015:15), there is little research in nursing literature that discusses intrapersonal skills, particularly in nursing education, whereas there is a rich supply of communication skills research and literature.

The concept of caring in nursing was a subject of intense interest in the latter decades of the 20th century (Bach & Grant 2015:25). However, very little literature is available as to whether soft skills training might be necessary for nursing institutions (Loffing 2005:725; Sills 2015:3). There is abundant literature on quality nursing, but not in the Ghanaian context. And there is very little documented on whether training in soft skills might help promote quality nursing care in Ghana.

### Purpose and objectives of the study

The study had the following objectives:

1. to explore nursing students’ understanding of the concept of soft skills in a selected nursing institution.
2. to explore the need for soft skills training among the nursing students.

## Methods

### Setting and recruitment

The study was conducted in one of the five nursing training colleges in the Upper East Region of Ghana. The population comprised the second- and third-year students of the registered general nursing (diploma) programme in the selected Nurses Training College, Bawku. The researcher selected this institution because it had students from all regions of Ghana.

### Respondents

The researcher used a sample size of 110 respondents for the questionnaire to obtain the data. According to Cohen, Manion and Morrison (2011:147), to obtain a representative sample in a population of 220 with a 90% confidence level and a 5% confidence interval, the estimated sample size is 115. Using a systematic random sampling calculation, the researcher divided the total number of students, 220, by 110. This resulted in a sampling interval of 2 being used as the constant difference between respondents (Polit & Beck 2010:316), with every second person on each class list being selected to participate, after the first respondent was randomly selected. Thus, the following formula was used to obtain the sampling interval: population/sample = sample interval = (220/110 = 2).

Data collection was carried out between 05 October and 20 October 2015, with a response rate of 99%. The researcher collected the data personally at the selected institution after obtaining permission.

## Analysis

Data were generated, organised and analysed using the Statistical Package for the Social Sciences (SPSS) Version 23. Descriptive statistics were used to calculate the mean and mode. The primary source used for data collection was a questionnaire.

### Ethical consideration

Ethical approval for this study was obtained from the College of Health Sciences of the University of KwaZulu-Natal Research Ethics Committee with ethical clearance number HSS/0350/015H. When this was secured, permission to proceed with the study was granted by the Principal of the selected Nurses Training College. All respondents were given information sheets, after which informed consent forms were signed and students were assured of their privacy and confidentiality during and after the study.

## Results

A total of 110 questionnaires were handed out to the respondents of which 109 were returned, giving a 99% response rate. The majority of respondents (62) were male (56%) and 65 (59%) were Christian. Also, 105 respondents (95%) were aged between 20 and 35 years as compared to only 6 (5%) who were aged below 20 years. Seventy-six respondents (69%) understood the concept of soft skills and agreed with the definition of soft skills. They furthermore agreed that soft skills should be part of the training that student nurses receive during their professional education because 62 respondents (55%) agreed that teaching soft skills in the classroom would help them to care well.

Similarly, 64 respondents (58%) believed that there is a need to train nurses in soft skills because this will improve the quality of nursing care and enhance professional performance.

More than half of the respondents, that is 76 students (69%), agreed that it was wrong to use offensive language, and to use bed numbers to refer to patients. They said these were inappropriate behaviours in the profession.

## Discussion

The objectives of the study were (1) to explore the nursing students’ understanding of the concept of soft skills and (2) to explore their perception of the need for soft skills training among nursing students. A quantitative research design with descriptive and explorative strategies was used in this study in order to determine the registered general nursing students’ understanding of the concept of soft skills and their perception of its use in training nurses to provide quality patient care.

### Understanding of the concept of soft skills

For the purposes of gaining students, perceived understanding of the concept of soft skills as non-technical skills that an individual possesses, the results suggested that the nursing students had adequate clues on what soft skills are, because a majority (98%) of the nursing students strongly agreed with the definition. The results indicated that acquisition of soft skills can make a career, while the absence of soft skills can break a career, to which most of the respondents (89%) strongly agreed. These findings are in line with a study by Prodanovic (2014:7), who contends that soft skills are key to lifelong learning because they are related to core competencies that deal effectively with demands and challenges encountered in daily life in every sphere of human endeavour. Pearson and McLafferty (2011:399) echoed this sentiment, arguing that understanding what non-technical skills or soft skills are and their relevance for healthcare practitioners has become a new area of consideration.

### The need for soft skills training in nursing

Regarding the need for training student nurses in soft skills, almost all respondents (92%) strongly agreed that there is a need to train student nurses in soft skills to help them gain the necessary non-technical skills needed. Students contend that soft skills are necessary skills which the nursing profession needs. These findings correlate with Pearson and McLafferty’s ideas (2011:399), which emphasise the necessity of introducing non-technical skills training and assessment within medical education, arguing that nursing education is yet to fully embrace this skills training. Similarly, in the health sector, training in soft skills is imperative because with expanding immigration, increasing globalisation and increased population growth, there is a need to enrich the diversity within the nursing profession, especially to better meet the needs of our changing society (Bednarz et al., 2010:253).

The results indicated that teaching soft skills in the classroom will help enhance the care rendered to clients. The majority (90%) of respondents strongly agreed with this fact. The results further suggested that soft skills training will enable nursing staff to give better care to their clients. This finding is similar to a study conducted by Dalaya et al. (2015:19), who stressed the importance of soft skills. They stated that soft skills increase confidence, professionalism, coordination, friendliness and optimism in an individual and that these attributes are significant in the nursing profession. They maintained that a combination of technical expertise and soft skills is very important for patient management and concluded that soft skills are essentially required for professionals, students, employees and clinical practitioners.

Furthermore, Deepa and Seth (2013:7) believe that the need to provide training in soft skills is seriously being considered today in most sectors, and they argue that soft skills mirror the ability to communicate and interact with others. These skills are unique because they emphasise action and so have become indispensable for every person in the present context.

### The need for soft skills training to promote quality nursing care

The study suggested that majority of respondents (95%) strongly agreed that soft skills training in nursing institutions might promote quality nursing care in health facilities. The students believed that training in soft skills will enhance their knowledge on patient care. This result is in agreement with a study conducted by Deepa and Seth (2013:8), who believe that insufficiency of soft skills in the nursing profession might be a precursor to situations where at certain times nurses are seen to seemingly ignore certain patients or are seen not to care. This is because they are ill-equipped emotionally by way of their training. Similarly, Rangnekar (2011:108) agreed with this, stating that most of the educational inputs deal with hard skills. For instance, the nursing education covers technology and techniques of giving injections, taking blood pressure measure, taking blood samples for medical tests, etc. However, the successful practice in each of these areas involves ‘soft skills’.

Furthermore, the study revealed that 95% of students strongly agreed that an individual with soft skills can enhance his or her professional interaction and job performance. This finding is in agreement with the study by Jain and Anjuman (2013:32), who state that most organisations invest a great deal of time and effort in elaborating training programmes designed to improve soft skills. This demonstrates companies understand the value of soft skills and their impact on their organisations. Today, in the world of employment, business executives consider soft skills a very important attribute in job applicants according to Jain and Anjuman’s findings.

Comparing hard skills and soft skills, Wats and Wats (2009:1) argued that there is enough literature that suggests hard skills contribute to only 15% of one’s success while the remaining 85% is made by soft skills. This explains why recognition of the importance of soft skills in today’s workforce has gained increasing momentum, with educators and industry bodies believing that generic skills are vitally important to business success (Osman, Girardi & Paull 2012:49).

## Recommendations

Nursing is a dynamic interpersonal process which seeks to promote, maintain and restore health. It is a unique enterprise whose practitioners are skilled in the assessment, planning, implementation and evaluation of health care. To meet the changing pattern of health care demands, nursing requires innovation to offer creative responses while working within an ethical and legal framework (NMC Ghana 2007:4). Based on the reviewed literature and the results, this research recommends the following: nursing education and recommendations for further research.

### Nursing education

The educational curriculum should include soft skills modules, and it should be assessed practically before registration for practice.

Educators and service providers should collaborate and work closely in reviewing and identifying learning needs. This should be coordinated by the staff development committee annually so that what is lacking in current nurses might be incorporated into training.

### Recommendations for further research

Further research is needed to acquire nurse educators’ and nurse practitioners’ perception of soft skills training in the nursing institutions and the need to incorporate soft skills into the nursing curriculum for training nurses in Ghana.

## Limitations of the study

Data collection was confined to registered general nursing students of a selected nursing institution in northern Ghana. Other institutions in southern Ghana could also have been sampled but were not because of lack of funds. Furthermore, nurse educators were not included. Such information could have added clarity about nursing students’ perception regarding soft skills training in nursing.

## Conclusion of the study

The findings of this research indicate that nursing students who participated in this study understood the basic concept of soft skills; however, they lacked a formal knowledge of what it entails. The study results further indicate that there is a need for nursing students to be educated in soft skills and that this will enhance their job performance in the clinical environment and improve the way they communicate with their clients.
